# The role of hypoxia in stem cell regulation of the central nervous system: From embryonic development to adult proliferation

**DOI:** 10.1111/cns.13754

**Published:** 2021-11-24

**Authors:** Gaifen Li, Jia Liu, Yuying Guan, Xunming Ji

**Affiliations:** ^1^ Laboratory of Brain Disorders Ministry of Science and Technology Collaborative Innovation Center for Brain Disorders Beijing Institute of Brain Disorders Capital Medical University Beijing China; ^2^ Department of Neurosurgery Xuanwu Hospital Capital Medical University Beijing China

**Keywords:** dentate gyrus, hypoxia, neurogenesis, stem cell, subventricular zone

## Abstract

Hypoxia is involved in the regulation of various cell functions in the body, including the regulation of stem cells. The hypoxic microenvironment is indispensable from embryonic development to the regeneration and repair of adult cells. In addition to embryonic stem cells, which need to maintain their self‐renewal properties and pluripotency in a hypoxic environment, adult stem cells, including neural stem cells (NSCs), also exist in a hypoxic microenvironment. The subventricular zone (SVZ) and hippocampal dentate gyrus (DG) are the main sites of adult neurogenesis in the brain. Hypoxia can promote the proliferation, migration, and maturation of NSCs in these regions. Also, because most neurons in the brain are non‐regenerative, stem cell transplantation is considered as a promising strategy for treating central nervous system (CNS) diseases. Hypoxic treatment also increases the effectiveness of stem cell therapy. In this review, we firstly describe the role of hypoxia in different stem cells, such as embryonic stem cells, NSCs, and induced pluripotent stem cells, and discuss the role of hypoxia‐treated stem cells in CNS diseases treatment. Furthermore, we highlight the role and mechanisms of hypoxia in regulating adult neurogenesis in the SVZ and DG and adult proliferation of other cells in the CNS.

## Graphical abstract

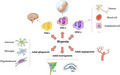

## 1 INTRODUCTION

Oxygen is a fundamental element for all living organisms and provides energy by taking part in metabolism.^1^ Hypoxia refers to oxygen levels lower than the normal value in the body or environment, which causes a series of different physiological and pathological reactions.^2^ The brain is the most sensitive organ to oxygen fluctuation. In particular, severe hypoxia usually causes acute and chronic brain damage; however, moderate hypoxia, such as levels used in intermittent hypoxia (IH) treatment, shows neuroprotective effects in various central nervous system (CNS) disease models.^3^, ^4^ Therefore, targeting hypoxia may be a potential therapeutic strategy for neurological diseases.^5^, ^6^ In this review, we focus on the roles and mechanisms of hypoxia in stem cells related to CNS, including embryonic stem cells (ESCs) and neuronal stem cells (NSCs), and also discuss the research progress of hypoxia in stem cell therapy of CNS diseases.

## 2 HYPOXIA RESPONSE

The body can respond to oxygen fluctuations through a series of molecular mechanisms, of which hypoxia‐inducing factors (HIFs) play a key role.^7^ HIF‐1, composed of HIF‐1α subunit and HIF‐1β subunit, is the most important transcription factor in response to hypoxia in the brain. Under normoxic conditions, the HIF‐1α subunit can be hydroxylated by proline hydroxylase, after which it binds with Von Hippel–Lindau complex to be ubiquitinated and degraded through proteasomes. Under hypoxia conditions, however, HIF‐1α cannot be degraded, but combines with HIF‐1β and translocates to the nucleus, promoting the transcriptional activation of multiple downstream hypoxia‐related genes, including vascular endothelial growth factor (VEGF), erythropoietin (EPO), glucose transporter 1, and more than 100 other genes.^8^

## 3 HYPOXIA AND STEM CELLS

Stem cells possess self‐renewal activities and multipotency, characteristics that tend to be maintained under hypoxic microenvironments.^9^ HIF signaling is a primary modulator for cellular metabolism in stem cells to maintain their undifferentiated status and pluripotent potential.^10^ In particular, stem cell niches usually exist in a hypoxic environment; for example, the oxygen level of the ESC niche is 2%–8%, and that of neural stem cells is 1%–8%.^11^ Hypoxia also maintains stem cell pluripotency and improves survival.^12^ In brief, hypoxia determines the fate of both embryonic and adult stem cells, as well as induced pluripotent stem cells (iPSCs) in vitro.^13^

### 3.1 Hypoxia and ESCs

Physiological hypoxia plays an important role in embryonic development and is involved in angiogenesis and blood flow regulation neural development, among others.^14^ Therefore, hypoxia treatment is thought to be beneficial to the culture of ESCs. In vitro experiments have shown that a hypoxic environment promotes stem cell survival; for example, 4% O_2_ maintains self‐renewal characteristics and limits the spontaneous differentiation of human ESCs (hESCs).^15^ Furthermore, 2%–5% O_2_ was shown to increase the total cell number approximately twofold compared with 20% O_2_ in mouse ESC cultures.^16^ Hypoxia was also found to facilitate the differentiation of ESCs into nerve cells. Under physiological hypoxic conditions, mouse ESCs were induced to differentiate into neural progenitor cells (NPCs), which were similar to mouse brain‐derived NSCs in terms of proliferation and self‐renewal ability, gene expression profile, and pluripotency.^17^ In addition, hypoxia stimulation promoted ESCs to differentiate into nerve cells but did not change the cell fate; early passaged ESCs tended to give rise to neurons, whereas late‐passaged ESCs tended to give rise to glial cells.^18^

Critical signaling molecules are involved in the regulation of ESCs under hypoxic conditions. Specifically, hypoxia was shown to promote pluripotency of hESCs and maintain their self‐renewal characters by promoting Notch activation.^19^ Hypoxia was also shown to regulate self‐renewal of hESCs through HIF‐2α and glycolytic sensors C‐terminal binding proteins.^20^ Hypoxic preconditioning enhanced hESC neural differentiation and cell survival by upregulation of HIF‐1α and HIF‐2α signaling.^21^ In addition to HIF‐mediated signaling, hypoxia also modulates ESCs via mitogen‐activated protein kinase (MAPK)/extracellular regulated protein kinase signaling.^22^ Taken together, these findings suggest that hypoxia is necessary to maintain the pluripotency and self‐renewal capacity of hESCs.

### 3.2 Hypoxia and NSCs and NPCs

Similar to ESCs, oxygen concentration is also involved in the regulation of NSCs and NPCs. NSCs and NPCs can self‐renew and generate terminally differentiated nerve cells that integrate into the neural circuitry and further contribute to the regulation of neurological function throughout life.^23^ During early cortical development, the presence of HIF‐1α prevents NSCs from producing differentiated progeny.^24^, ^25^ Furthermore, during embryonic neural development, the effects of hypoxia on NSCs are temporally regulated. Specifically, hypoxia inhibits NSC differentiation and maintains their undifferentiated state during early development, whereas it induces neural differentiation at later stages.^26^ Recently, NSC‐based treatment was shown to be a promising therapeutic strategy in hypoxic‐ischemic brain injury, which is an important cause of morbidity and mortality in adults and newborns. The above data indicate that hypoxia promotes the differentiation and development of iPSCs, which may have a promising therapeutic outcome in CNS diseases.

Multiple signaling pathways are involved in the regulation of NSCs by hypoxia. Hypoxia was shown to increase the proliferation of NSCs by upregulating HIF‐1α expression and activating the Wnt/β‐catenin pathway.^27^ Similarly, hypoxia‐induced HIF‐1α expression prevented NSCs from premature neuronal differentiation by activating neural repressor Hes1, which is independent of Notch signaling.^28^ Hypoxia also upregulated the expression of several HIF‐1α downstream proteins, including VEGF.^29^ In addition, hypoxia promoted cell proliferation by increasing miR‐21 expression in NSCs; an action possibly mediated by phosphatidylinositol 3‐kinase (PI3K) signaling pathway activation.^30^ Finally, hypoxic exposure was found to promote proliferation of NPCs via PI3K/protein kinase B (AKT)‐dependent glycogen synthase kinase‐3β signaling.^31^

Hypoxia and ischemia triggered NPC proliferation by upregulating complex 1‐chromobox7 through HIF‐1α activation.^32^ Ischemia and hypoxia by unilateral carotid occlusion also promoted migration and proliferation of NPCs through chemokine upregulation.^33^ RNA‐binding protein RBM3 was found to be highly upregulated in response to hypoxia, which in turn increased proliferation of primary NSCs.^34^ In addition, gene set enrichment analysis identified the calcium‐regulated transcription factor NFATc4, which is significantly upregulated in NSCs after hypoxia treatment, as a potential candidate in the regulation of hypoxic NSC functions.^35^ Therefore, hypoxia plays an important role in the development and differentiation of NSCs and NPCs and suggests that understanding the molecular mechanism of hypoxia‐mediated behavioral changes in NSCs and NPCs is helpful to optimize stem cell therapies for neurological diseases.^36^, ^37^

### 3.3 Hypoxia and iPSCs

Advances in human iPSC technology have resulted in the development of new drug candidates for many CNS diseases by capturing patient heterogeneity. Hypoxia may enhance iPSC generation and maintenance. Specifically, iPSC generation has been shown to be poor under normoxic conditions; however, under mildly hypoxic conditions (5% O_2_), iPSC generation and reprogramming efficiency increased.^38^ The pluripotency of iPSCs is a prerequisite for their differentiation and expansion, and hypoxic conditions help to maintain the pluripotency of iPSCs.^39^ In addition, hypoxia was shown to promote iPSCs to differentiate into specific cell types, such as endothelial cells (ECs),^40^, ^41^ neurons,^42^, ^43^ and cardiomyocytes.^44^ HIF‐1α signaling is critical for iPSC pluripotency and lineage differentiation.^45^ In addition, FGFR1‐induced activation of PI3K/AKT and MAPK signaling was also involved in mild hypoxia‐mediated maintenance of iPSC pluripotency.^46^

### 3.4 Hypoxia and stem cell treatment

Due to the poor regeneration capacity of the nervous system, stem cell transplantation therapy is a promising strategy for CNS disease treatment.^47^ Mesenchymal stromal cells (MSCs) are a group of cell types commonly used in stem cell therapy.^48^, ^49^ These cells are distributed in various tissues, such as bone marrow, umbilical cord, nasal mucosa, and fat; furthermore, their pluripotency is largely regulated by hypoxia.^50^ Hypoxia has regulatory effects on cell vitality and repair effects on cell function. MSCs cultured under hypoxic conditions upregulated several stem cell markers and promoted cell proliferation.^51^, ^52^ Exosomes derived from hypoxia‐treated MSCs promoted functional behavioral recovery in a spinal cord injury mouse model by shifting microglial polarization from the M1 to M2 phenotype.^53^

Bone MSCs (BMSCs) are commonly used stem cells, as they have been shown to develop into neurons and glia in vitro. Hypoxic preconditioning of BMSCs enhanced generation of NPCs^54^ and the secretion of bioactive factors.^55^ Interestingly, a combination of hypoxia and modest inflammatory stimuli promoted the migration of BMSCs.^56^ Mechanically, hypoxia preconditioning enhanced BMSC survival and reinforced their regenerative properties by upregulating HIF‐1α and various trophic/growth factors, including brain‐derived neurotrophic factor (BDNF), VEGF, and EPO. Hypoxia also promoted the proliferation and migration of umbilical cord blood‐derived human MSCs via the HIF‐1α/FASN/mTORC1 axis.^57^ Hypoxia preconditioning enhanced BMSC survival after transplantation by activating HIF‐1α in a spinal cord injury model. Transplantation of these hypoxia pretreated BMSCs enhanced neurogenesis and angiogenesis in cerebral ischemia rats.^58^ In addition, hypoxic conditions were also shown to affect other types of stem cells, for example, the proliferation of MSCs in adipose tissue of livestock and their differentiation and transformation into pluripotent stem cells.^59^ Furthermore, treatment with human amnion epithelial cells could alleviate hypoxic‐ischemic injury in the perinatal brain.^60^ Hypoxic preconditioning increased grafted‐cell survival of NSCs and improved therapeutic effects of NSC transplantation in a hemorrhagic stroke mouse model.^61^ Finally, hypoxia‐preconditioned olfactory mucosa MSCs were shown to inhibit the death of microglia after cerebral ischemia/reperfusion insult via HIF‐1α activation.^62^

### 3.5 Hypoxia and adult neurogenesis

Neural stem cells exist not only in the developing mammalian nervous system but also in the adult nervous system. Among them, the lateral ventricle subventricular zone (SVZ) and hippocampal dentate gyrus (DG) are recognized as the most concentrated regions of NSCs in the adult brain.^63^ Under certain conditions, NSCs can differentiate into neurons to participate in the repair process of nerve function, which is called neurogenesis.^64^ The biggest difference between the two regions is that DG NSCs cannot be transported over a long distance, whereas those of the SVZ can; therefore, SVZ NSCs are a better model for studying neural cell value‐added migration and differentiation.^65^ Similar to embryonic neurodevelopment, in adult mammals, moderate hypoxia can also promote neurogenesis both in the SVZ and DG.^66^

### 3.6 Hypoxia and SVZ neurogenesis

**FIGURE 1:**
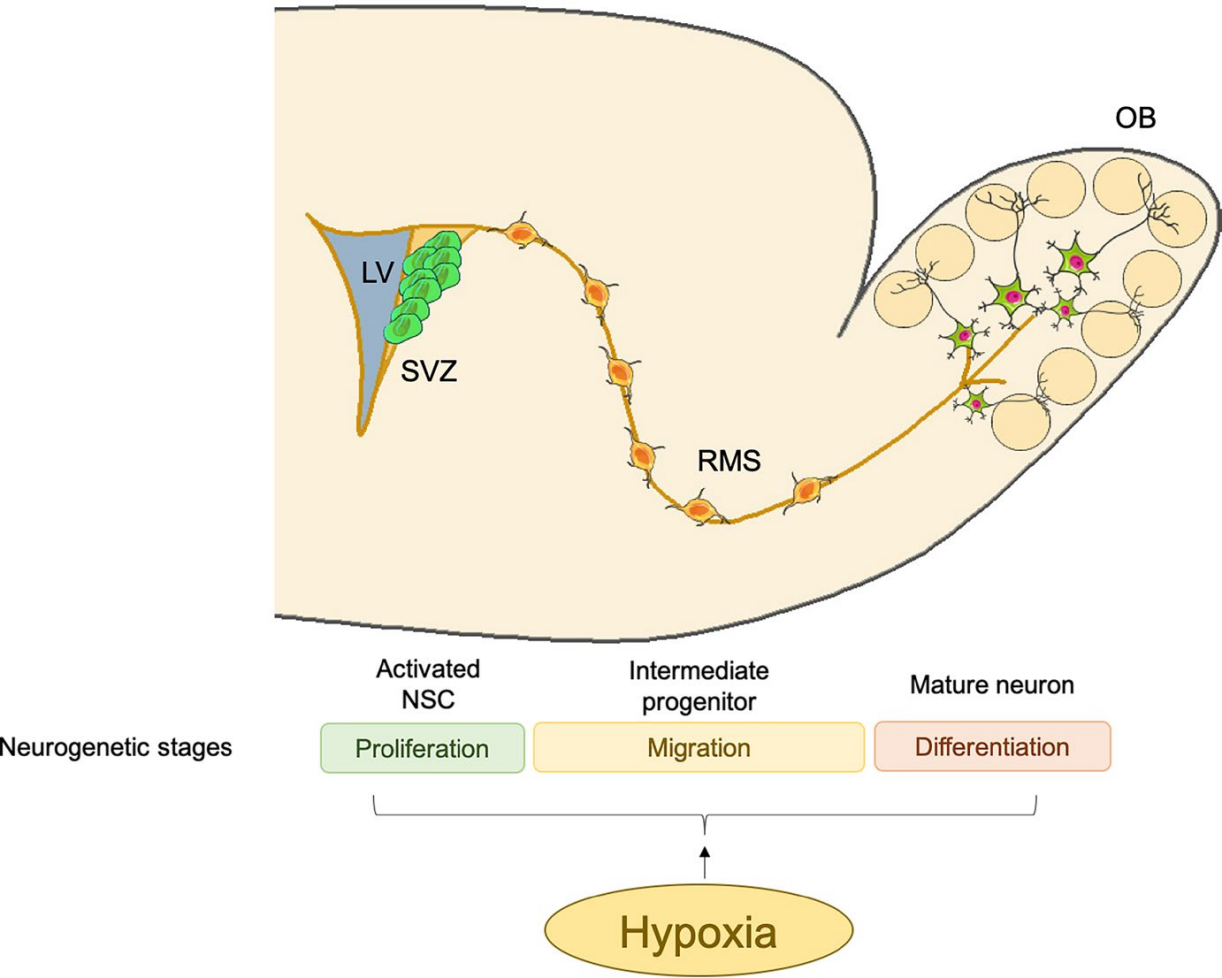
Hypoxia involves subventricular zone (SVZ) neurogenesis. Neural stem cell (NSC) in lateral ventricle (LV) SVZ could migrate a long distance to the olfactory bulb (OB) through rostral migratory stream (RMS). Hypoxia promotes SVZ neurogenesis in several stages, including proliferation, migration, and differentiation

Hypoxia is involved in SVZ neurogenesis throughout life (Figure 1). In fact, most of the neuroblasts produced from SVZ NSCs migrate a long distance to the olfactory bulb where they differentiate into local neurons.^67^ Hypoxia and hypoxic preconditioning were shown to enhance the regenerative capacity of neural progenitors in the perinatal SVZ region.^68^, ^69^ Furthermore, perinatal asphyxia promoted cell proliferation and neurogenesis in the SVZ, delayed cell death and affected the neural circuits of the basal ganglia and hippocampus, although the mechanism remains to be elucidated.^70^ Neonatal hypoxic‐ischemic injury also promoted SVZ neurogenesis.^71^ IH enhanced expansion and differentiation of NPCs in the SVZ.^72^ Creating an endogenous hypoxic environment, such as during intense exercise, restored the normal cell cycle length and quiescent phase of stem cells and neuroblasts by promoting the proliferation of adult SVZ stem cells of mice.^63^

At the molecular level, HIF‐1α signaling is necessary for the maintenance of NSCs and vascular stability in the SVZ. In particular, genetic inactivation of HIF‐1α results in gradual loss of NSCs in the adult SVZ.^73^ As hypoxia has been shown to upregulate the expression of HIF‐1α and VEGF and to promote the regulation of cell division in the SVZ,^74^ it may also improve the prognosis of newborns after ischemia and hypoxia.^75^ H19, a long noncoding RNA, was significantly upregulated during the hypoxic response of SVZ NSCs in a focal cerebral ischemia rat model.^76^

### 3.7 Hypoxia and hippocampal DG neurogenesis

**FIGURE 2:**
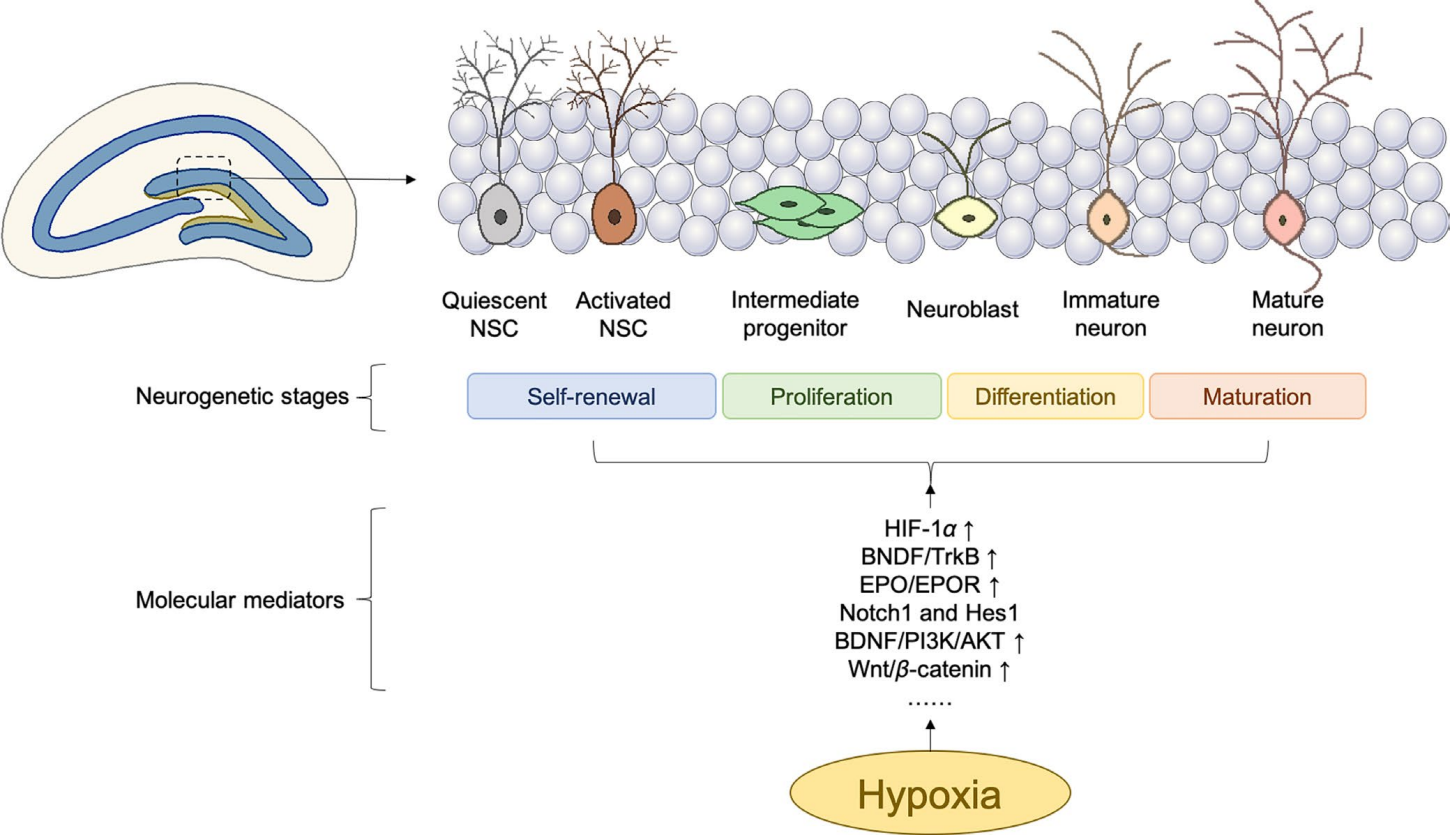
Hypoxia involves hippocampal neurogenesis. Hypoxia promotes hippocampal neurogenesis in various stages, including self‐renewal, proliferation, differentiation, and maturation. Several mediators or pathways are involved in these processes, such as HIF‐1α, brain‐derived neurotrophic factor (BDNF)/tryrosine receptor kinase B (TrkB), erythropoietin (EPO)/EPO receptor (EPOR), and so on. AKT, protein kinase B; PI3K, phosphatidylinositol 3‐kinase

The hippocampus is characterized by the presence of lifelong neurogenesis (Figure 2).^77^, ^78^ Specifically, newborn neurons in the hippocampus are mainly generated by dentate granule cells, which then integrate into the neural circuit and maintain hippocampal function.^79^ Many adult newborn cells die during early differentiation in the hippocampus, with oxidative damage being a critical factor.^80^ Similar to SVZ NSCs, hypoxia also determines the survival and proliferation of newborn cells derived from the DG.^81^, ^82^

Adult hippocampal neurogenesis is critical for cognitive function, especially during aging or after brain injury, such as ischemia.^83^ Newborn hippocampal neurons contribute to memory performance by establishing functional synapses with target cells.^84^ Hippocampal neurogenesis in DG improve cognitive and emotional remodeling in chronic unpredictable mild stress‐induced rats.^85^ A moderate hypoxic treatment was shown to enhance adult hippocampal neurogenesis both in vitro and in vivo.^4^, ^86^ Furthermore, mild hypoxic conditions increased NSCs proliferation, promoted newborn neuron survival and migration, and contributed to the maturation of hippocampal neurons.^81^, ^87^ Therefore, activation of neurogenesis by hypoxic treatment may be a potential therapeutical strategy for various CNS diseases.^88^

Chronic hypoxia (10% O_2_) treatment was shown to stimulate hippocampal neurogenesis by activating the Wnt/β‐catenin signaling pathway in an Alzheimer's disease mouse model.^89^ Chronic intermittent hypobaric hypoxia also rescued spatial and object memory deficits by promoting hippocampal neurogenesis and synaptic plasticity via the Wnt/β‐catenin pathway in pilocarpine‐treated epileptic rats.^90^ IH, a type of mild hypoxia strategy, is thought to have neuroprotective effects in many types of CNS diseases.^91^ A study showed that IH rescued spatial learning and prevented memory impairment by inducing hippocampal neurogenesis.^92^ IH was also found to alleviate long‐term memory impairment induced by ischemic injury by increasing hippocampal neurogenesis and synaptogenesis via BDNF/PI3K/AKT signaling.^92^ IH enhanced NSC proliferation, newborn neurons survival, and dendritic spine morphogenesis in the DG by activating Notch1, whereas Notch1 deficiency inhibited hippocampal neurogenesis induced by IH.^87^ HIF‐1α–Notch signaling is also involved in increased neurogenesis in epilepsy rats.^93^ Consecutive mild hypoxia exposure for 28 days was shown to contribute to hippocampal neurogenesis and rescue cognitive deficits by activating Notch1 and Hes1 signaling in epileptic rats.^94^

Brain‐derived neurotrophic factor is a neurotrophic factor involved in neurogenesis and neuroplasticity.^95^, ^96^ Severe hypoxia was shown to inhibit synaptic plasticity, which contributed to cell death and impaired neurogenesis.^97^ However, modest episodes of hypoxia were found to exert neuroprotective effects by triggering adaptation of cells.^98^, ^99^ Enhancement of BDNF expression is regarded as a recognized protective mechanism of hypoxia‐related treatment, such as IH. Specifically, IH improved cognitive function and depressed anxiety by enhancing BDNF expression in the hippocampus of mice.^4^ Furthermore, IH was found to produce antidepressant‐like effects by targeting BDNF‐tyrosine receptor kinase B signaling in rats.^81^ In addition, IH promoted serotonin‐dependent BDNF synthesis and improved synaptic plasticity.^100^ Post‐ischemia IH intervention increased synaptogenesis via upregulation of BDNF in neurons^92^ and protected vulnerable neurons from hypoxia/ischemia‐induced injury.^101^ Meanwhile, enhancing BDNF secretion from brain ECs into the cerebral microvasculature has also been considered as a potential therapeutic target of IH treatment.^102^ IH training combined with physical exercise promoted the proliferation of endogenous neural progenitors and further enhanced BDNF expression in the adult hippocampus, eventually leading to an increased number of newborn neurons.^103^ Taken together, these results suggest that moderate IH treatment exerts neuroprotective effects by promoting BDNF expression and secretion.

## 4 HYPOXIA AND NON‐NEURONAL CELL PROLIFERATION IN THE CNS

Hypoxic responses in the CNS are not limited to stem cell proliferation; other cells also undergo changes in this hypoxic environment,^104^ such as vascular ECs^105^ and glial cells,^106^ among others. Hypoxia‐induced changes can promote metabolism, which is conducive to energy production and waste excretion in the body.^107^ At the same time, hypoxic conditions can provide energy for signal transmission, such as neurogenesis and stem cell proliferation, which are significant for CNS subordinate feedback loops that help to adjust the subordinate to initiate corresponding strategies through the integration of information; the CNS in turn sends signals to instruct the subordinate to initiate the corresponding changes.^108^ Glial cells are significant components of the CNS and, as they do not conduct electrical impulses like neurons, have long been considered to play a supporting role. It is only in recent years that scientists have begun to realize the regulatory role of glial cells in the brain.^109^ In the following sections, we discuss the effects of hypoxia on glial cells and ECs from the perspective of cell proliferation.

### 4.1 Hypoxia and astrocytes

Astrocytes are the most widely distributed type of cells in the mammalian brain and are also the largest type of glial cells.^110^ They are star‐shaped with many long and branched protrusions from the cell body, which stretch and fill the space between neuronal cell bodies and their projections, helping to support and separate neurons.^111^ In particular, because astrocytes are involved in the structural and functional interface of cerebral circulation and neuronal networks, they play an important role in the resistance to hypoxia‐induced brain damage.^112^

Hypoxia can stimulate astrocytes to activate and proliferate to help maintain neuronal networks and cerebral microcirculation.^113^ Astrocytes are able to regulate cerebral blood flow to maintain constant PO_2_; furthermore, local astrocytes are also involved in the protective effect of hypoxia‐induced ischemic nerve injury.^112^ Hypoxia can induce the proliferation of astrocytes via various pathways, such as HIF^114^ and Notch.^115^ Hypoxia‐induced astrocyte proliferation is involved in the formation of glial scarring following ischemic hypoxic brain injury.^116^, ^117^ Hypoxic preconditioning can upregulate glucose transporter levels and activity in astrocytes during acute hypoxia, increasing glucose uptake and promoting cell glycolysis and lactic acid production, which provide energy for neuronal activity.^118^, ^119^, ^120^

### 4.2 Hypoxia and microglia

Microglia are considered to be the largest population of local immune cells in the CNS and are involved in the removal of damaged neurons, plaques, and infectious substances.^121^, ^122^ However, microglia play a “double‐edged sword” role in CNS diseases, as studies have shown that abnormal microglial activation is involved in the pathogenesis of neurodegenerative diseases, such as Parkinson's disease, multiple sclerosis, and Alzheimer's disease.^123^, ^124^, ^125^

Different degrees of hypoxic treatment have different effects on residential microglia or repopulated microglia; the underlying mechanisms remain obscure. In general, mild and short‐term hypoxic stimulation activates resident microglia first,^126^, ^127^, ^128^ whereas stronger continuous hypoxia induces microglia proliferation. Microglia are considered to be the final product of neural differentiation, a special support cell that is very different from neurons, whereas recent studies suggested that some glial cells may act as primary progenitors or NSCs.^129^ These glial cells could differentiate and proliferate under hypoxia stimulation and exert neuroprotective effects.^104^, ^130^ Specifically, hypoxia treatment produced mildly stressed microglia, which activated a feedback loop to regulate the increase in antiinflammatory factors, thus exerting a protective effect. Therefore, hypoxia treatment not only causes mild stress in microglia to modulate inflammatory responses but also stimulates the differentiation and proliferation of certain microglia to promote CNS recovery following an insult.

### 4.3 Hypoxia and oligodendrocytes

Oligodendrocytes, which are smaller than astrocytes, are widely distributed in the CNS. Their main function is to surround axons to form an insulating myelin structure and to further assist in the efficient transmission of bioelectrical signals.^131^

Oligodendrocyte progenitor cells (OPCs) are the main proliferative cells in the adult brain, which differentiate into myelinated oligodendrocytes during the CNS development.^132^ When the nervous system is damaged, especially myelin‐related damage, OPCs differentiate and proliferate to aide in repair.^133^, ^134^ Hypoxia and HIF signaling play an important role in stimulating OPCs differentiation.^135^ HIF signaling activated‐patient‐derived iPSCs transplanted into the brain during the subacute phase of white matter stroke induced the proliferation and remyelination of endogenous oligodendrocyte precursors and promoted axonal budding and, thus, cognitive recovery effects.^136^ HIF‐1α‐induced oligodendrocyte lineage gene‐1 expression promotes the growth of oligodendrocytes and triggers the repair of hypoxic‐induced neuronal myelin damage.^137^

### 4.4 Hypoxia and vascular ECs

Vascular proliferation refers to the formation of new blood vessels by sprouting or intussusception from preexisting blood vessels through the proliferation and migration of ECs.^138^ This process is critical for the transportation of oxygen and nutrients to cells through the blood, which in turn promotes tissue regeneration, development, and repair.^139^ Recent evidence suggests that there may be an interaction or dependence between stroke‐induced neurogenesis and angiogenesis. Ischemic stroke promotes neurogenesis through growth factors such as VEGF and improves the recovery of neurological function after stroke.^140^ VEGF increases the proliferation of NSCs through the VEGF receptor 2 signaling pathway, promotes the migration of new cells, actively participates in the initial stage of neurogenesis, and reduces cognitive impairment after epilepsy.^141^ Vascular ECs play a significant role in regulating blood pressure and the balance of coagulation and anticoagulation, so as to maintain the normal flow of blood and the long‐term patency of blood vessels.^142^ VEGF is highly specific to vascular ECs and has important biological functions such as promoting the proliferation of ECs. VEGF is a downstream gene of HIF, and its expression is upregulated by hypoxic stimulation.^143^ In the developing brain, neurons expressing VEGF‐A are closely linked to blood vessels, and miR‐9 directly targets the transcription factors TLX and ONECUTs to regulate VEGF expression. Due to the dual role of miR‐9 in the proliferation and angiogenesis of neural stem cells, the regulation of VEGF expression by miR‐9 is of great research significance for the treatment of stem cell regeneration.^144^ Neurovascular unit (NVU) is a special cerebrovascular structure composed of ECs, neurons, pericytes, glial cells, and other cells; it plays an important role in maintaining brain function.^145^ At present, there are few studies on the relationship between hypoxia and NVU; in‐depth and systematic researches on this field are of great significance for us to understand the role of hypoxia‐induced neurogenesis and angiogenesis in treatment of CNS diseases.

## 5 PERSPECTIVES AND CONCLUSION

Hypoxia plays a significant role in the construction of the microenvironment and differentiation of NSCs and in the protection of neurons after CNS injury. NSCs have the characteristics of self‐renewal and the ability to differentiate into multiple cells and, as such, have broad application prospects in cell transplantation therapy.^146^ Hypoxia was shown to enhance the directional differentiation of stem cells. Furthermore, hypoxia promoted ion human iPSCs to differentiate into NSCs by regulating the Wnt/β‐catenin pathway. Together, these findings have important implications for potential therapeutic strategies for CNS diseases.^147^ At present, NSC transplantation is a hot topic in the treatment of stroke,^148^ spinal cord injury,^149^ traumatic brain injury,^150^ and other CNS diseases. Indeed, NSCs were shown to induce the release of Mir‐133b in BMSCs to promote the survival of neurons, thus further improving the therapeutic effect of NSC transplantation on cardiac arrest‐induced brain injury.^151^ In recent years, hypoxia has been proven to induce stem cell differentiation, which indicates that hypoxia combined with stem cell therapy is not only a therapeutic target for ischemic stroke but also shows great potential for multiple therapeutic strategies in other CNS diseases treatment. However, further studies are needed to understand the molecular mechanisms of hypoxia in the treatment of CNS diseases.^152^

In addition to CNS diseases, hypoxia combined with stem cell therapy can be applied to a variety of other diseases. Hypoxia promoted human iPSCs to differentiate into neural crest cells, which produced functional EPO and induced hematopoietic progenitor cells differentiate into erythrocytes, thus exerting therapeutic effects in renal and nonrenal anemia.^153^ In addition, hypoxia may have a role in delaying senescence, and a previous study showed that the life span of mammalian primary cells increased under hypoxia conditions.^154^ It has also been shown that hypoxia can induce MSCs to differentiate into tendon, which indicates that hypoxia combined with stem cell therapy could also be applicable to tendon injury intervention.^155^ Taken together, the results indicate that hypoxic treatment combined with stem cell therapy can be applied to combat anemia, aging, tendon injury, and so on. However, the molecular mechanisms associated with this combined therapeutic approach are still unclear, which limits the application of these therapies.

**FIGURE 3:**
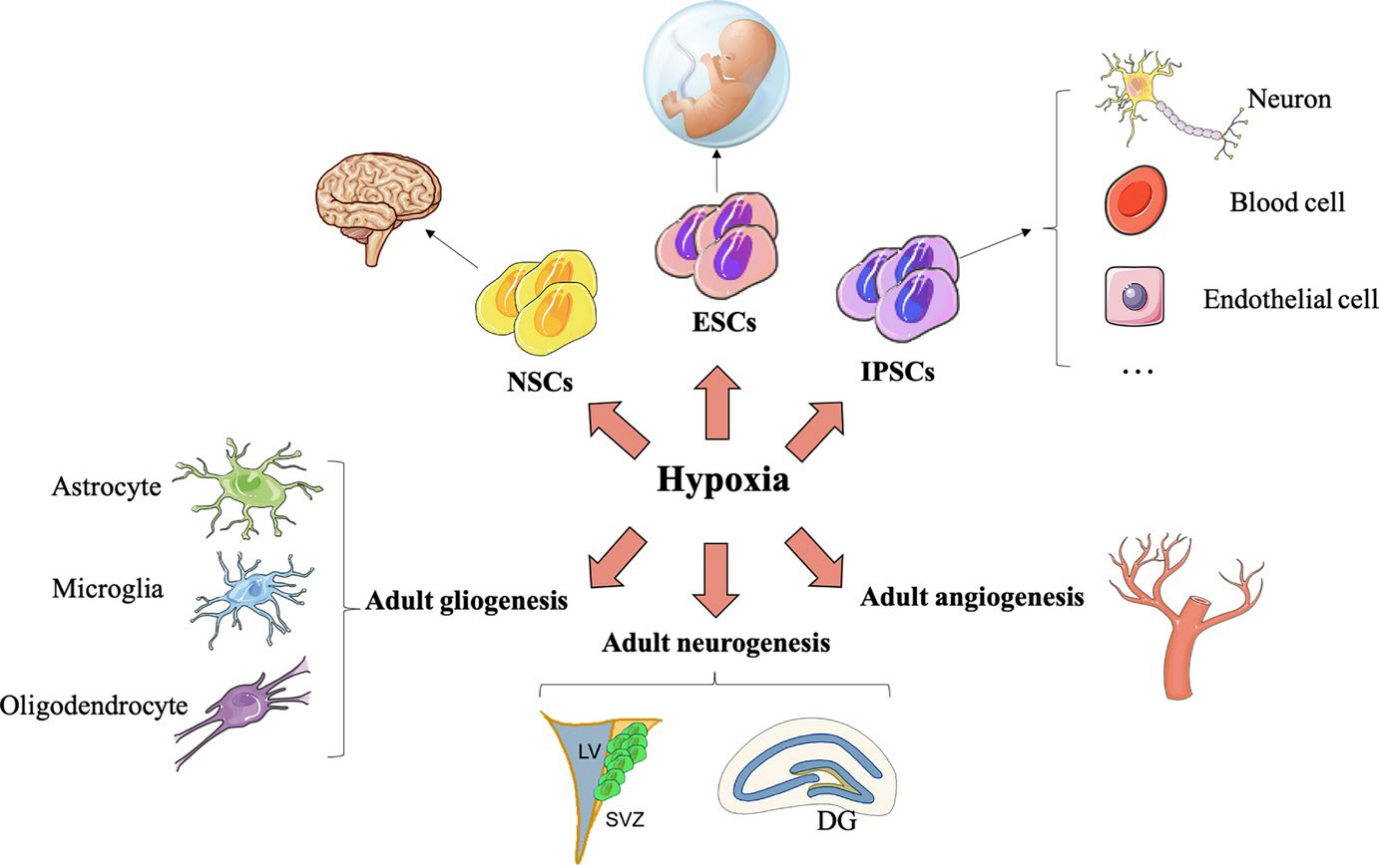
Hypoxia promotes stem cell proliferation, differentiation, and survival. Hypoxia promotes the pluripotency, proliferation, and directed differentiation of embryonic stem cells (ESCs) and neural stem cells (NSCs) and helps induced pluripotent stem cells (iPSCs) to develop into different types of cells in vitro. Hypoxia is also involved in adult neurogenesis, angiogenesis, and gliogenesis. DG, dentate gyrus; LV, lateral ventricle; SVZ, subventricular zone

In conclusion, hypoxia can induce many types of stem cells to proliferate, differentiate, and develop into specific cell types, such as neurons. Although its specific molecular mechanisms are not entirely clear yet, hypoxic treatment has great therapeutic promise for the treatment of CNS diseases. Therefore, it is important to further investigate the molecular mechanisms of hypoxia‐induced stem cell maintenance and optimize and promote the use of hypoxia in stem cell therapy associated with CNS diseases (Figure 3).

## CONFLICT OF INTEREST

The authors declare that they have no competing interests.

## Data Availability

Data sharing does not apply in this article because no new data were created or analyzed in this study.
